# Pediatric supracondylar humeral fractures: Does pin and splint removal 3 weeks after surgery increase the risk of secondary displacement?

**DOI:** 10.1007/s00590-026-04985-7

**Published:** 2026-09-30

**Authors:** Andreas Tsoupras, Viola Sbampato, Johanne Mazurié, Ardian Ramadani, Giacomo De Marco, Romain Dayer, Oscar Vazquez, Dimitri Ceroni, Anne Tabard-Fougère, Christina Steiger

**Affiliations:** Pediatric Orthopedic and Traumatology Unit, University Hospital of Geneva, Geneva, Switzerland

**Keywords:** Supracondylar humeral fractures, Pediatric, Fracture fixation, Postoperative complications, Displacement, Pin-tract infection, Immobilization

## Abstract

**Purpose:**

Supracondylar fractures of the distal humerus are among the most common pediatric fractures. For unstable fractures, reduction with pin fixation and postoperative immobilization is standard, but the optimal duration of pin fixation and splinting remains uncertain. This study evaluated whether pin and splint removal approximately 3 weeks after surgery was associated with secondary fracture displacement in children treated for displaced supracondylar humeral fractures.

**Methods:**

We retrospectively reviewed pediatric patients surgically treated for displaced supracondylar humeral fractures at our institution between January 2015 and December 2024. Both closed and open reduction cases were eligible. Collected data included demographics, Rigault-Lagrange fracture stage, fixation construct, duration of pin and splint immobilization, radiographic displacement before and after removal, and postoperative complications.

**Results:**

A total of 211 patients were included (mean age 6.5 ± 2.3 years). According to the Rigault-Lagrange classification, 87 fractures were stage 2, 54 were stage 3, and 70 were stage 4. Pins and splints were removed after a mean of 23.6 ± 4.0 days. Secondary radiological displacement was observed in 11 patients, corresponding to 13 displacement events: 10 occurred before pin removal and 3 after removal. Three patients showed a change in Baumann’s angle greater than 12° after removal, and no patient lost intersection of the anterior humeral line with the capitellar ossification center after removal. One patient required early revision before pin removal, and no patient required delayed corrective osteotomy.

**Conclusion:**

In this retrospective cohort, pin and splint removal approximately 3 weeks after surgical fixation of pediatric supracondylar humeral fractures was associated with a low rate of subsequent secondary displacement, irrespective of fracture stage. Most displacement events occurred before pin removal, and no patient required delayed corrective osteotomy. Early removal may limit exposure to complications associated with prolonged pin fixation and immobilization, although this potential benefit requires confirmation in comparative studies.

**Level of evidence:**

III (retrospective cohort study).

## Introduction

Supracondylar fractures of the distal humerus (SCH) are among the most common pediatric fractures, representing approximately 15% of all childhood fractures and 48–78% of elbow fractures [1–3]. Management ranges from cast immobilization for stable, nondisplaced fractures to closed reduction and pin fixation for unstable but reducible injuries. Open reduction with pinning is generally reserved for fractures associated with soft-tissue entrapment, vascular injury, or failed closed reduction [4, 5]. Common constructs include two lateral retrograde pins, crossed fixation with one medial and one or two lateral pins, or three lateral pins. After fixation, the elbow is immobilized in a splint to enhance stability. Pins may be left exposed to outpatient removal with appropriate analgesia or buried, requiring a second procedure under general anesthesia [6].

Although adequately treated supracondylar fractures generally have good to excellent functional outcomes [3], complications may still occur and can adversely affect long-term elbow function. The most reported treatment-related complications include secondary displacement (2.9–13.4%), pin migration, pin-tract infection, postoperative stiffness, and malunion with recurvatum, cubitus varus or valgus with tardy ulnar nerve palsy [3, 4, 7–13].

However, there is still no consensus on the optimal timing of pin removal [2, 14–16]. Ideally, pins should be removed neither too early, as this may compromise fracture stability, nor too late, as this may unnecessarily prolong immobilization, limit joint motion, and increase the risk of stiffness and pin-tract infection [17].

Clinicians may remain hesitant to remove pins and splints early, particularly after displaced or unstable fractures. Evidence specifically addressing secondary displacement after early, simultaneous removal of both pins and postoperative immobilization remains limited.

The primary objective of this study was to assess whether pin and splint removal approximately 3 weeks after surgery was associated with secondary fracture displacement in children treated for displaced supracondylar humeral fractures. Secondary objectives were to characterize the timing of displacement, assess displacement according to fracture stage and fixation construct, and describe associated complications and clinical outcomes. We hypothesized that early pin and splint removal would be associated with a low rate of subsequent secondary displacement, irrespective of fracture stage.

## Methods

### Study design and setting

This retrospective cohort study was conducted at a tertiary university hospital from 2015 to 2024 and reported according to the Strengthening the Reporting of Observational Studies in Epidemiology (STROBE) guidelines. The study was approved by the institutional ethics committee (CCER no: 2017-01617) and all the subjects or their legal representant provided written informed consent for the use of their data in research.

### Population

Patients who underwent pin fixation for displaced supracondylar humeral fractures were identified from institutional electronic medical records using diagnostic and procedural codes. The following inclusion and exclusion criteria were applied:

Eligibility criteria: [1] Children younger than 16 years with a supracondylar fracture of the distal humerus; [2] Surgical treatment and follow-up completed at the institution; [3] At least 3 months of clinical and radiographic follow-up; and [4] Full adherence to postoperative treatment principles during follow-up. Both closed and open reduction cases were eligible for inclusion. The absence of a palpable radial pulse at presentation was not an exclusion criterion.

Exclusion criteria: [1] Previous fracture or surgery involving the ipsilateral elbow; [2] Pathological fracture; [3] Bilateral supracondylar fractures; [4] Floating elbow injury pattern; [5] Surgery performed at another hospital; [6] Follow-up shorter than 3 months; [7] Incomplete medical records or radiographic data; [8] Radiographs of insufficient quality because of excessive artefacts; [9] Non-adherence to postoperative treatment principles during follow-up.

Sources and methods for participants selection: All eligible patients meeting inclusion criteria during the study period were identified through retrospective chart review. Patients were followed longitudinally with standardized assessments at baseline and follow-up visits (minimum three-months interval).

### Surgical technique and follow-up protocol

All patients were treated by board-certified, fellowship-trained pediatric orthopaedic surgeons. Fixation primarily involved two divergent lateral pins. Fracture stability was assessed intraoperatively, and an additional pin was inserted when residual instability persisted. The choice between a third lateral pin and a medial crossing pin depended on the level and orientation of the fracture line and the ability to obtain adequate fixation. Whenever sufficient stability could be achieved with an additional lateral pin, this configuration was preferred over medial pin insertion. Postoperatively, all patients were immobilized in a posterior plaster splint with the elbow at approximately 90° of flexion. The splint was assessed during postoperative follow-up and was maintained without routine conversion to a circumferential cast if it remained well fitted and undamaged. Patients were scheduled for pin and splint removal at approximately 3 weeks after surgery. After pin removal, radiographs were obtained to confirm fracture alignment, immobilization was discontinued, and progressive elbow mobilization was initiated. Additional routine follow-up was performed at 6 weeks, 3 months, and thereafter as clinically indicated. Physiotherapy was not routinely prescribed, except in cases of neurological impairment or persistent limited mobility 12 weeks after surgery.

### Variables and data sources

Medical records were reviewed to extract data on patient demographics (age and sex), injury characteristics (Rigault-Lagrange fracture type and neurovascular status at injury), surgical details (pin number, fixation construct, and any additional fracture treatment), and postoperative outcomes (time to pin and splint removal and complications).

### Fracture classification and radiographic assessment

Fractures were classified according to the Rigault-Lagrange system [18]. Reduction quality and post-removal displacement were evaluated on radiographs obtained immediately after surgery, before pin removal at 3 weeks, and after pin removal. On lateral views, the position of the anterior humeral line (AHL) relative to the capitellar ossification center was recorded [19].

On anteroposterior views, the epiphyseal-diaphyseal angle (Baumann’s angle) was measured, normal values ranging from 64° to 81°, with a mean of 72–75°.

### Primary and secondary outcomes

Based on prior literature, a change greater than 12° between two radiographs was considered significant and served as the primary outcome [20, 21] for the coronal plane. On the sagittal plane, significant displacement was defined by AHL passing anterior to the capitellar ossification center, with no intersection [22, 23]. Secondary outcomes included systematically recorded complications, such as pin-tract infection [24], symptomatic malunion requiring reoperation, and nerve injuries involving the radial, ulnar, or median nerve.

### Sample size calculation

Before the study, a power analysis was performed for secondary fracture displacement, based on an expected rate below 5% from previous studies [10, 25, 26]. The final cohort of 211 patients exceeded the required sample size of 203, which provided 80% power with a ± 3.0% margin at the 95% confidence level.

### Statistical analysis

Analyses were performed using R software (version 4.4.1, R Foundation for Statistical Computing, Vienna, Austria) and the RStudio interface (version 2024.09.0, Posit software, PBC). The level of significance was set at *p* < 0.05. The distribution of continuous variables was assessed using the Shapiro-Wilk test. Comparisons of continuous variables across the three Rigault-Lagrange fracture stages were performed using the Kruskal-Wallis test when the assumption of normality was not met. Associations between categorical variables, including secondary displacement and fracture stage, were assessed using Fisher’s exact test because of the small number of displacement events.

Descriptive statistics were calculated for all variables. Continuous variables were reported as mean ± standard deviation [range] or median [interquartile range], as appropriate, and categorical variables as n (%). When an association between two continuous variables was assessed without assuming normality, Spearman’s rank correlation was used.

## Results

### Population flow and recruitment

**Fig. 1:**
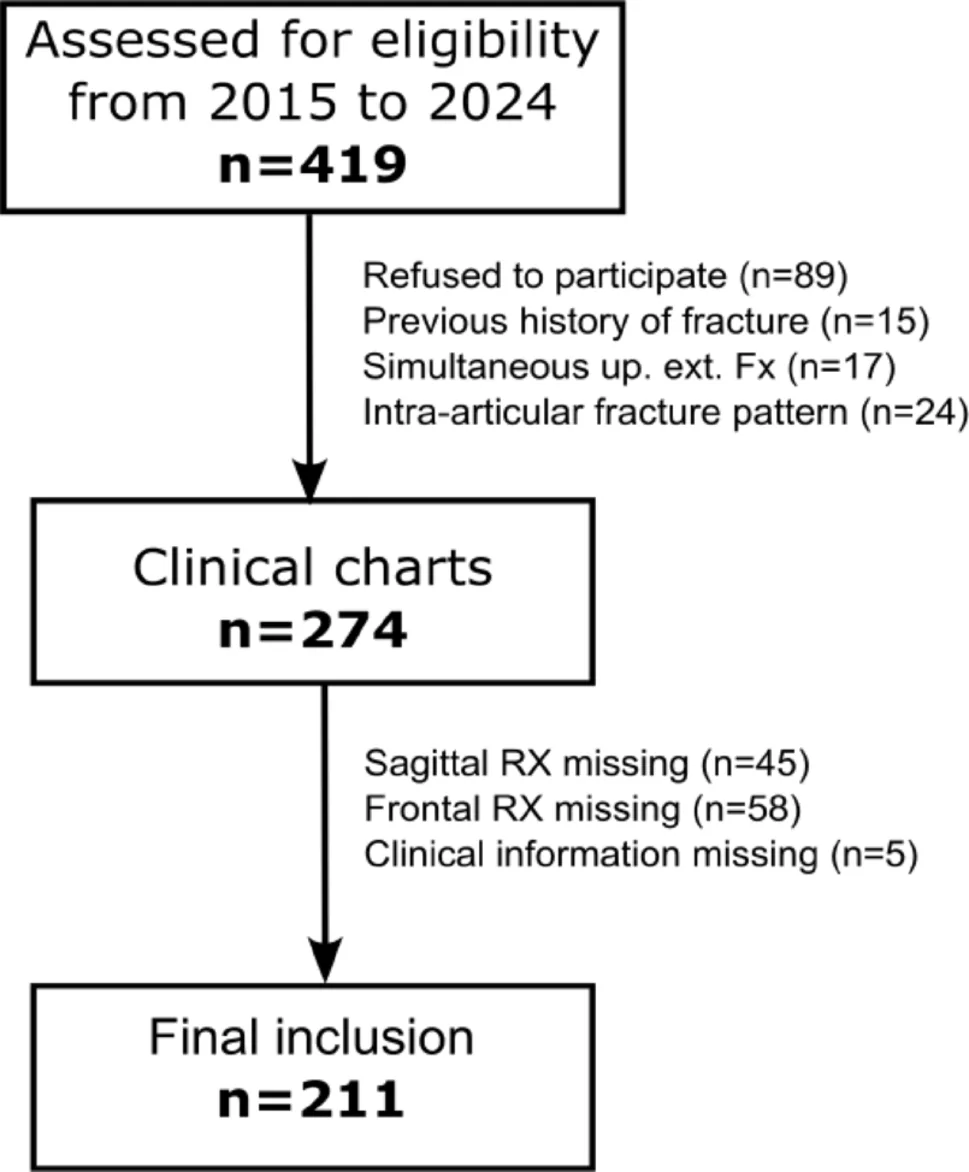
Flow chart of the included patients

The cohort included 211 patients with supracondylar distal humeral fractures (108 males and 103 females), all with at least 3 months of follow-up. The mean age was 6.5 ± 2.3 years (range, 1.0–12.9). The right side was affected in 42% of cases. Seven patients (3.3%) presented without a palpable radial pulse; five had a well-perfused hand despite the absence of a palpable pulse, whereas two presented with a pale, ischemic hand. The patient selection flowchart is shown in Fig. 1.

### Fracture and surgical treatment characteristics

Table 1 summarizes patient epidemiological characteristics and fracture stages: 87 patients had Rigault-Lagrange stage 2 fractures, 54 had stage 3 fractures, and 70 had stage 4 fractures. Most patients underwent closed reduction and percutaneous pinning (206/211, 98%); 5 patients required open reduction. Fixation consisted of two lateral pins in 161 cases, three lateral pins in 30 cases, and crossed pins (two lateral and one medial) in the remaining 20 cases (Table 2). Pins and splints were removed after a mean of 23.6 ± 4.0 days after surgery.

**Table 1:** Epidemiological data of the 211 included patients

Outcomes	Values
Mean age ± SD (years)	6.5 (2.3) [range: 1.0–12.9]
Male sex, n (%)	108/211 (51.2%)
Season	
Spring n (%)	63/211 (29.9%)
Summer n (%)	59/211 (28.0%)
Autumn n (%)	65/211 (30.8%)
Winter n (%)	24/211 (11.4%)
Rigault-Lagrange stage	
Type II, n (%)	87/211 (41.2%)
Type III, n (%)	54/211 (25.6%)
Type IV, n (%)	70/211 (33.2%)

Data are presented as mean (standard deviation) [range] for continuous variables and n (%) for categorical variables.

**Table 2:** Operative parameters and complications

Outcomes	Values
Pin construct type	
2-lateral pins	161/211 (76.3%)
3-lateral pins	30/211 (14.2%)
Crossed pins	20/211 (9.5%)
Displacement during follow-up	11/211 (5.2%)
Surgical revision for secondary displacement	1 (0.4%)
Corrective osteotomy (n)	0 (0.0%)
Pin track infections	4/211 (1.9%)

Data are presented as n (%) for categorical variables.

### Secondary displacement

Secondary radiological displacement was observed in 11 patients with a total of 13 displacements; 10 displacements occurred before pin removal and 3 after removal (2 patients had displacements before and after on the coronal plane). Displacement occurred in the sagittal plane in 2 cases and in the coronal plane in 11 cases. Secondary displacement did not appear to be associated with fracture severity (Table 3).

**Table 3:** Secondary fracture displacement data

Outcomes	Before pin removal*	After pin removal*
Displacement per fracture stage		
Type II, n (%)	3/87 (3.4%)	0/87 (0%)
Type III, n (%)	3/54 (5.5%)	0/54 (0%)
Type IV, n (%)	4/70 (5.7%)	3/70 (4.3%)
Displacement per construct type		
2-lateral pins	9/161 (5.6%)	1/161 (0.6%)
3-lateral pins	1/30 (3.3%)	2/30 (6.7%)
Crossed pins	0/20 (0%)	0/20 (0%)
Direction of displacement		
Frontal plane, n (%)	8/13 (61.5%)	3/13 (23.1%)
Sagittal plane, n (%)	2/13(15.4%)	0/13 (0.0%)
Number of displacements (*n* = 13)	10/13 (76.9%)	3/13 (23.1%)

*Secondary radiological displacement was observed in 11 patients with a total of 13 displacements (2 patients had displacements before and after on the coronal plane). 10 displacements occurred before pin removal and 3 after removal

### Timing and severity of displacement

Analysis of displacement timing showed that 10 of 13 displacement events (76.9%) occurred before pin removal and 3 (23.1%) after removal. Displacement according to fixation construct is presented in Table 3.

### Other complications

Four children developed infectious complications. Two had pin-tract infections associated with septic arthritis of the elbow and required pin removal, surgical debridement, arthrotomy with irrigation, and antibiotic therapy. The other two were treated with pin removal and antibiotics alone. All infections resolved without long-term sequelae. Neurological deficits were observed in 14 patients: 8 had anterior interosseous nerve palsy (57%), 2 had ulnar nerve palsy (14%), and the remaining patients had radial nerve palsy. Both patients with ulnar nerve palsy underwent closed reduction and fixation with two lateral pins, without medial pin insertion. One ulnar nerve deficit was documented preoperatively, whereas the second was identified postoperatively; in the latter case, the preoperative neurological examination was limited by pain and fracture deformity, precluding definitive determination of whether the deficit was pre-existing or related to the reduction procedure. All patients achieved full functional and clinical recovery within 6 to 12 months, and none required secondary nerve surgery.

### Clinical outcomes

One patient needed early surgical revision one week postoperatively, treated with secondary closed reduction and pinning. This patient presented with major coronal plane displacement and had uneventful outcomes at 3 months postoperatively. None of the included patients required delayed corrective osteotomy for distal humeral realignment. Functional outcomes were considered satisfactory during follow-up, including in patients with posterior displacement and recurvatum of up to 20°. The two patients with displacement on the sagittal plane, were 4 and 5 years old respectively and showed satisfactory remodeling during follow-up. In the coronal plane, changes in Baumann’s angle were not associated with clinically significant varus or valgus deformity or impaired elbow function.

## Discussion

The present study evaluated a standardized protocol of pin and splint removal approximately 3 weeks after surgical fixation of displaced pediatric supracondylar humeral fractures. Only 3 of 13 displacement events occurred after pin removal, whereas the majority occurred while fixation was still in place. No patient required delayed corrective osteotomy. These findings suggest that, when satisfactory stability has been maintained until the 3-week assessment, simultaneous removal of pins and splint is associated with a low risk of clinically relevant subsequent displacement.

Previous studies have also reported low rates of loss of reduction, although postoperative protocols and removal timing vary. Aubret et al. [27] observed no secondary displacement after K-wire removal at a mean of 29.8 days; the two early displacements in their series occurred before removal and were attributed to inadequate initial reduction or fixation. Pennock et al. [26] reported a 4.2% loss-of-reduction rate, whereas Yaokreh et al. [28] reported a 3.4% rate of secondary displacement after K-wire removal at 6 weeks. In a survey of European Paediatric Orthopaedic Society members, most respondents reported pin removal at 3–4 weeks after surgery [29]. Jones et al. [2] found that a mean immobilization period of 28.8 days achieved good outcomes without increased complications compared with longer immobilization, while prolonged immobilization has been associated with poorer short-term elbow motion [17].

The present findings also showed no clear association between fracture stage and secondary displacement. This is relevant because more displaced fractures are often presumed to require longer immobilization. Pennock et al. similarly found that fracture type and several patient and fracture characteristics were not associated with loss of reduction [26]. Nevertheless, the small number of displacement events in the present cohort limits definitive subgroup conclusions.

The timing of displacement is also clinically important. Most displacement events in this cohort occurred before pin removal, supporting the importance of initial reduction and fixation stability. Technical factors associated with early loss of fixation include inadequate pin purchase and insufficient pin separation at the fracture site [10, 25]. Available evidence suggests that delayed displacement after pin removal is uncommon when alignment has remained stable until removal [30].

Radiographic displacement did not necessarily translate into clinically relevant impairment. One patient required early revision before pin removal, but no patient underwent delayed corrective osteotomy. The two sagittal displacement cases showed satisfactory remodeling during follow-up. These observations should be interpreted cautiously because validated patient-reported functional outcomes were not systematically available.

Early discontinuation of percutaneous fixation and immobilization may also reduce exposure to pin-related infection and postoperative stiffness. The pin-tract infection rate in this cohort was 1.9%. However, because there was no comparison group with prolonged pin fixation or immobilization, the present study cannot establish that removal at 3 weeks reduces infection or stiffness; these should therefore be considered potential rather than demonstrated benefits.

This study has several limitations inherent to its retrospective design. Data were collected from records of variable quality, and cases with incomplete data were excluded. Potential confounders such as physical activity, bone quality, and contralateral elbow measurements were not available. Radiographic measurement variability may have resulted from differences in image acquisition and positioning. The cohort was drawn from a single center, and no control group with prolonged immobilization was available. Finally, the limited number of secondary displacement events precluded robust subgroup analyses or identification of independent risk factors.

Future multicenter comparative studies should directly evaluate removal at approximately 3 weeks versus longer immobilization using secondary displacement, pin-tract infection, validated functional outcomes, range of motion, and reoperation as predefined outcomes.

## Conclusion

In this retrospective cohort, pin and splint removal approximately 3 weeks after surgical fixation of pediatric supracondylar humeral fractures was associated with a low rate of subsequent secondary displacement, irrespective of fracture stage. Most secondary displacement events occurred before pin removal, and no patient required delayed corrective osteotomy. These findings support the safety of early pin and splint removal in appropriately stabilized fractures, while the potential benefits regarding pin-tract infection and stiffness require confirmation in comparative studies.

## Data Availability

Data of the current study are not publicly available. They are, however, available upon request.

## Abbreviations

SCH: Supracondylar fractures of the distal humerus
AHL: Anterior humeral line
